# The Role of Nutrition in HPV Infection and Cervical Cancer Development: A Review of Protective Dietary Factors

**DOI:** 10.3390/cancers17183020

**Published:** 2025-09-16

**Authors:** Maria Guitian, Gabriel Reina, Silvia Carlos

**Affiliations:** 1 School of Pharmacy and Nutrition, Universidad de Navarra, 31008 Pamplona, Spain; mguitianval@alumni.unav.es; 2 Microbiology Department, Clínica Universidad de Navarra, 31008 Pamplona, Spain; 3 IdiSNA, Navarra Institute for Health Research, 31008 Pamplona, Spain; scarlos@unav.es; 4 Department of Preventive Medicine and Public Health, Universidad de Navarra, 31008 Pamplona, Spain

**Keywords:** cervical cancer, human papillomavirus, nutrition, diet

## Abstract

While nutrition is often considered a protective factor against cancer, there is a scarcity of strong, conclusive evidence supporting its direct impact on cervical cancer (CC) prevention. This study aims to review the existing literature on the relationship between women’s nutrition and infection by the human papillomavirus (HPV) and the development of CC. It explores the key nutrients, foods, and dietary patterns that may influence the onset and progression of this disease, including research carried out in sub-Saharan Africa, other low-income countries, and worldwide. The review process revealed that, despite the large initial pool of articles, the majority were excluded because they were not focused on nutrition and CC. However, the articles that met the inclusion criteria suggested an association between diet and lower susceptibility to persistent HPV infections, which could progress into cervical lesions or cancer. Notably, lower folate intake was associated with up to a nine-fold higher risk of CIN3+ lesions (OR 8.9, 95% CI 3.4–24.9), while increased consumption of certain vitamins and antioxidant-rich foods consistently correlated with lower HPV persistence and progression. These results could indicate that consuming antioxidant compounds, certain vitamins, dietary patterns, and functional foods may offer protective benefits against developing this disease and its progression. By emphasizing nutrition as a modifiable factor, this review seeks to raise awareness and propose a holistic approach to CC prevention—integrating public health, dietary considerations, and equity-driven strategies.

## Simple Summary

There is a scarcity of evidence supporting nutrition impact on cervical cancer (CC). This study reviews the literature on the association between women’s nutrition (nutrients, foods and dietary patterns), human papillomavirus (HPV) infection, and CC development, including research carried out in sub-Saharan Africa and other low-income regions. Antioxidants, certain vitamins, dietary patterns rich in fruits, and vegetables and functional foods may offer protective benefits against developing this disease and its progression. In contrast, diets with a high glycemic load or pro-inflammatory properties may contribute to disease progression. By emphasizing nutrition as a modifiable factor, this review seeks to raise awareness and propose a holistic approach to CC prevention—integrating public health, dietary considerations, and equity-driven strategies.

## 1. Introduction

Cervical cancer (CC) is the fourth most common cancer among women worldwide, both in incidence and mortality. In 2022, more than 650,000 new cases were reported globally, with approximately 20% occurring in Africa. Sub-Saharan Africa (SSA) bears a disproportionate burden of this disease, where CC is the second most prevalent cancer and causes over 80,000 deaths annually [1].

Persistent infection with high-risk types of Human Papillomavirus (hrHPV) is the necessary cause for CC [2]. However, HPV alone is not sufficient to cause cancer. Several cofactors—including smoking, long-term use of hormonal contraceptives, high parity, immunodeficiency, and, particularly, HIV infection—are known to increase the risk of HPV persistence and progression to cervical intraepithelial neoplasia (CIN) and invasive cancer [3,4]. Among the less explored cofactors, nutritional status and diet have been proposed as potentially significant but understudied contributors to CC development [5]. While a general link between nutrition and cancer risk is well-established, the evidence regarding its role in CC remains inconclusive. Studies suggest that micronutrients with antioxidant or anti-inflammatory properties—such as vitamins A, C, E, and folate—might enhance immune response and reduce oxidative stress, thereby reducing HPV persistence and DNA damage [6,7,8,9]. Nevertheless, the results are inconsistent, and no clear consensus exists on the effectiveness of specific dietary patterns in CC prevention. The recent literature raises the possibility that anti-inflammatory or antioxidant-rich diets could reduce susceptibility to HPV infection and disease progression, particularly in immunocompromised populations. However, few studies have directly examined these associations, especially in high-burden regions like SSA.

Despite advances in HPV vaccination and screening, many women—especially in low-resource settings—lack access to these preventive measures. Thus, identifying additional, low-cost strategies such as dietary modification could offer an important complement to existing interventions.

## 2. Methods

A comprehensive literature review was conducted to assess the association between women’s nutrition and the development of CC, with particular attention to nutrient intake and dietary patterns and geographic focus. The review includes peer-reviewed articles published up to March 2025 that addressed the role of diet, specific nutrients, food, or dietary patterns in relation to HPV persistence or CC. Studies were included if they provided original data or systematic reviews focused on this association. Articles were excluded if they focused exclusively on CC treatment, vaccination, or screening, or if they addressed other cancers or HPV-related malignancies without specific reference to CC.

The search was performed in PubMed using the MeSH terms “cervical cancer”, “nutrition”, “HPV”, “diet”, and “food”, combined in various ways using Boolean operators (AND, OR) to ensure a comprehensive search strategy. Additionally, filters were applied to identify existing reviews and to confirm that no recent detailed synthesis had already been published on this topic. Upon confirming the novelty of the review, a second phase of the search expanded the scope to other world regions and included more specific MeSH terms such as “Vitamin A”, “Vitamin E,” “Ascorbic Acid,” “Carotenoids,” “Vitamin B Complex,” “Iron,” “Food,” “Diet,” and “Prevention,” while still including “cervical cancer” and “Africa” to retain thematic focus, using the same Boolean operators. Articles identified in the initial searches were screened by title and abstract, followed by full-text review. Data was systematically extracted, focusing on study design, population characteristics, nutritional exposures, and clinical outcomes. During this process, references within eligible studies were also reviewed to identify additional relevant publications. All selected studies were analyzed, and relevant data were extracted and organized in summary tables according to nutrient type, food groups, dietary pattern, study design, population characteristics, and findings related to HPV or CC risk or progression. No new datasets, proprietary codes, or experimental protocols were generated for this study and all analyzed materials were publicly available from peer-reviewed sources.

## 3. Results

### 3.1. Articles Selected

**Figure 1:**
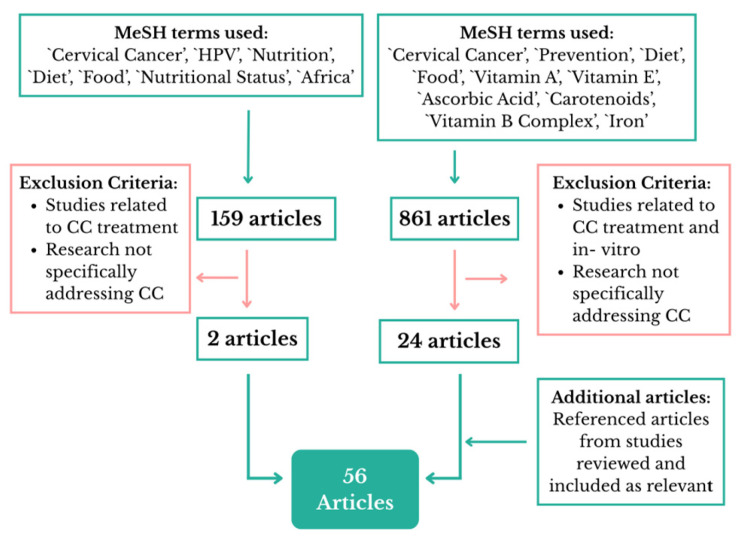
Flowchart of the literature reviewed. Created by the authors.

The initial search focused on SSA yielded a total of 159 articles, which were screened by reviewing their titles and abstracts (Figure 1). Those meeting the inclusion criteria were thoroughly analyzed; however, only two studies specifically addressed nutrition and CC in Africa.

The second search resulted in 861 articles. After checking their titles and abstracts, 72 articles were selected, of which 24 were finally used. Excluded articles were primarily omitted because, although they mentioned nutrition or CC, they did not provide specific data on the effect of nutrients or the role of diet in CC development.

When the term “Africa” was added to this last review, five articles were found, though only one complied with the inclusion criteria. Thus, the information was extracted from a total of 56 articles.

#### Characteristics of the Selected Studies

**Figure 2:**
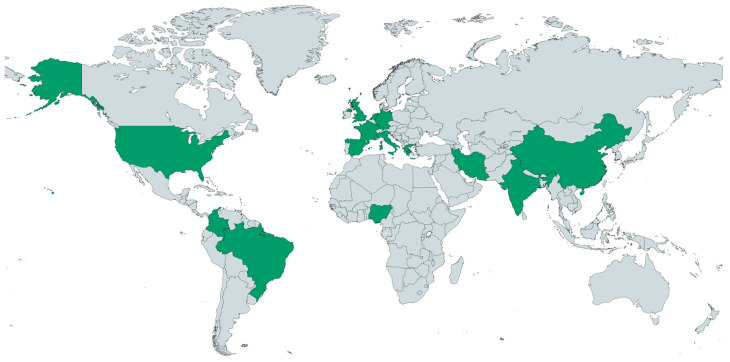
Map of countries where studies relating CC and nutrition have been conducted (in green). Created by the authors.

The vast majority of studies relating nutritional aspects to the development of CC were conducted in countries where the burden of this disease is lower (Figure 2). The lack of studies in the most-affected areas clearly highlights the gap in research on this topic.

Most studies were carried out in Asia (n = 19, 26%) (mainly in China) and the USA (n = 11 studies, 15% of the studies), and there were almost no studies from Africa and Europe and none from Oceania. This geographic concentration of studies may limit the generalizability of the findings to other regions, as dietary patterns, nutritional status, and CC risk factors can vary across populations. The studies mainly had a case–control or cohort design, although there were also some cross-sectional studies and clinical trials.

### 3.2. Diet and Cervical Cancer

**Figure 3:**
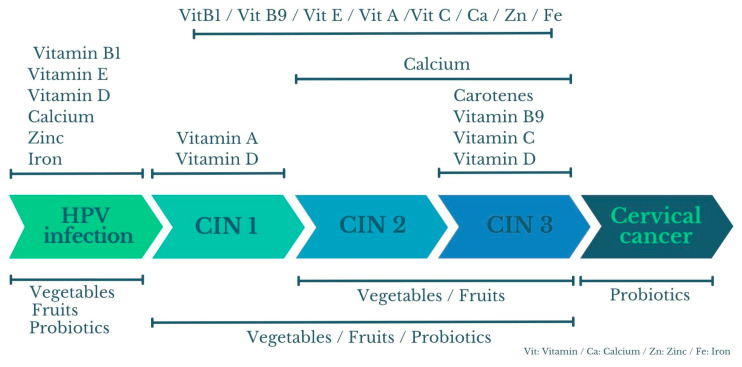
Nutrients and their influence on CC phases. The nutrient phase assignments illustrated in Figure 3 are based on trends observed in the included studies, as summarized in Table 1, Table 2, Table 3, Table 4, Table 5 and Table 6, and are meant to provide a conceptual overview rather than cite individual articles. Created by the authors.

The findings suggest that different nutrients exert an influence and/or action on different phases of disease progression (Figure 3).

#### 3.2.1. Water-Soluble Vitamins

Several studies identified a potential association between water-soluble vitamins—particularly B-complex vitamins and vitamin C—and a reduced risk of CIN and CC (Table 1).

A 2023 study from Iran found that vitamin B1 (thiamine), along with vitamins B3 and B6, was correlated with a lower risk of CC [10]. Additionally, an inverse association was reported between vitamin B1 supplementation (2 mg/day) and HPV infection rates [11]. Low serum levels of vitamin B1 were linked to increased CIN persistence and progression, as well as poor prognosis [12].

Regarding vitamin B9 (folate), multiple studies showed that lower folate levels were associated with an increased risk of CIN progression and persistence, potentially though impaired DNA methylation affecting gene regulation [12,13,14], while folate supplementation supported CIN regression [15]. In the U.S., a folate intake exceeding 433 µg/day was correlated with a decreased risk of CC [16].

Vitamin C intake also showed an inverse relationship with cervical neoplasia risk. Specifically, an intake greater than 225 mg/day was associated with reduced CC risk [16], and higher vitamin C levels were linked to a decreased risk of squamous cell cervical carcinoma [17]. Lower plasma levels of vitamin C were observed in women with CC in a case–control study [18] and were associated with CIN persistence and poor prognosis [12].

**Table 1:** Studies analyzing the effect of water-soluble vitamins on HPV infection, CC risk and/or progression, by date.

Ref.	Country	Year of Publication	Study Design Data Collection	Sample Size Population Age (Years) HIV/HPV+	Vitamin	Results
[10]	Iran	2023	Population-based cross-sectional study. Machine learning model	n = 2088 Mean age 34	Vitamin B1 Vitamin B3 Vitamin B6 Vitamin E	Vitamin–CC: Vitamin B1 (Thiamine) −0.558 (r) Vitamin B3 (Niacin (mg)) −0.648 (r) Vitamin B6 −0.602 (r) Vitamin E −0.730 (r)
[13]	Colombia	2023	Case–control study (nested in trial)	Cases CIN 2+ (n = 155) Controls ≤ CIN1 (n = 155) Age 20–69	Folate B9	Folate deficiencies–CIN3+ (affect DNA methylation): OR 8.9 (95% CI 3.4–24.9)
[14]	China	2022	Cohort study (cross-sectional analysis of baseline data)	n = 2304 Age 19–65	Folate (B9)	↓ serum levels of RBC folate–all CIN. ORs: Q1 vs. Q4: 2.28 (95% CI: 1.77, 2.93). Similar inverse associations for CIN1/2/3+. ↓ serum levels of RBC folate–progression CIN1 to CIN2: Q1 vs. Q4: 3.86; (95%CI:1.01, 14.76) ↑1-unit reduced risk of CIN1 progress to CIN2 (0.67; 95% CI: 0.46, 0.99)
[12]	China	2021	Prospective cohort. FFQ	N = 564 Age 18+	Vitamins B1; B3; B7; B9; C	Low levels–CIN persistence and progression: Vitamin B1 (RR 1.86) Vitamin B3 (Niacin) (RR 2.98) Vitamin B6 (RR 2.11) Vitamin B7 (Biotin) (RR 2.14) Vitamin B9 (Folate) (RR 15.22) Vitamin C (RR 2.19)
[11]	USA	2020	Cohort (NHANES) 24 h recall questionnaire	N = 13471 Age 18–59	Vitamin B1 (thiamine)	Vitamin B1–HPV infection: OR 0.70 (95% CI 0.63, 0.77). Best preventive effect with intake ≈ 2 mg. Excessive intake does not increase the preventive effect
[15]	Iran	2016	Randomized, Clinical trial	n = 49 Folate (n = 25) Placebo (n = 24) Ages 18–55	Folate (B9)	Folate supplementation (5 mg/d) vs. placebo (6 months) promotes CIN1 regression: 83% vs. 52%, (*p* = 0.019)
[17]	Europe *	2010	EPIC cohort	n = 299,651 ICS (n = 253) CIS (n = 817) Age 35–70	Vitamin C	Higher intake of vitamin C–invasive squamous cell CC. HR = 0.59 (95% CI 0.39–0.89)
[16]	USA	2007	Case–control study FFQ	Cases (n = 239) Controls (n = 979) Age 21–59	Folate (B9) Vitamin C	Folate intake > 433.2 μg/day–CC: OR = 0.55; (95% CI 0.34–0.88) Vit C intake > 224 g/day–CC: OR = 0.53; (95%CI 0.33–0.8)
[18]	India	2002	Case–control	Cases (n = 30) Controls (n = 30) Age 35–55	Vitamin C	Vitamin C plasma levels are lower in cases vs. controls

Table abbreviations: low (↓), confidence interval (CI); carcinoma in situ (CIS); red blood cells (RBC; Cervical Cancer (CC); food frequency questionnaire (FFQ); hazard ratio (HR); invasive squamous cervical cancer (ISC); odds ratio (OR); quartile (Q); risk ratio (RR); correlation coefficients (r). Values reflect different study designs and are not directly comparable. * Europe refers to the countries that participated in the EPIC cohort: France, Germany, Greece, Italy, The Netherlands, Spain, and the United Kingdom.

#### 3.2.2. Liposoluble Vitamins

Vitamin A is a liposoluble vitamin obtained through the diet and synthesized from carotene [19]. Table 2 shows that a low intake of vitamin A (retinol) has been associated with an increased risk of cervical intraepithelial neoplasia stage 1 (CIN1) [20]. Also, a higher intake of Vitamin A is strongly inversely associated with CC risk [21]. A U.S. study showed that consuming over 12.7 IU of vitamin A daily reduced the risk of CC by nearly 50% [16]. However, when referring to serum Vitamin A, a meta-analysis performed in 2022 revealed that high circulating vitamin A concentrations were not significantly connected with the risk of CC [22].

Vitamin E has also been inversely associated with HPV infection [23]. Higher α-tocopherol (vitamin E) intake was linked to a lower risk of CIN2/3 and invasive CC [24]. An intake greater than 8.9 mg/day of vitamin E was associated with a 66% risk reduction for CC [16]. In a case–control study, plasma vitamin E levels were lower in cases compared to controls [18], and lower serum α-tocopherol levels were associated with more severe cervical lesions [25].

Vitamin D deficiency is common, especially in women of reproductive age. In a clinical trial conducted in Iran, supplementing 50,000 IU of vitamin D every 14 days for 6 months led to increased CIN regression [26]. A cohort study also reported a protective effect of higher vitamin D intake against the development of squamous cell CC [17]. Additionally, lower serum vitamin D levels were associated with higher hrHPV prevalence [27]. These findings are clinically relevant, but supplementation should always be considered in consultation with a healthcare professional to ensure safety and appropriateness for each patient.

**Table 2:** Studies analyzing the effect of liposoluble vitamins on HPV infection and CC risk and/or progression, by date.

Ref.	Country	Year of Publication	Study Design Data Collection	Sample Size Population Age (Years) HIV/HPV+/−	Vitamin	Results
[10]	Iran	2023	Population-based cross-sectional study. (MLM)	n = 2088 Mean age 34	Vitamin E	Vitamin E–CC correlation: −0.730 (r)
[28]	China	2020	Cohort	n = 2304 Age 18–60+	Vitamin K	Vit–CIN2+ (for optimal dose): Q2 OR 1.53 (95% CI 1.02–2.29)
[23]	USA	2020	Cohort (NHANES)	n = 5809 Age 18–59	Vitamin E	Vitamin E–HPV infection (especially hrHPV) Q4 vs. Q1 OR 0.72 (95%CI 0.65–0.80)
[26]	Iran	2016	Randomized-placebo-controlled clinical trial	n = 58. Age 18–55, CIN1 diagnosis	Vitamin D	Supplementing 50,000 IU every 14 days 6 months was associated with a higher regression vs. placebo
[27]	USA	2016	Cross-sectional study	Age 18+, HPV+	Vitamin D	Decrease in serum Vit. D levels (per 10 ng/mL)-hrHPV prevalence OR 1.14 (95% CI 1.02–1.27)
[17]	Europe *	2010	Prospective cohort study	n = 299,651 ICS (n = 253). CIS (n = 817) Age 35–70	Vitamin D	Higher intake–invasive squamous cell CC. HR = 0.47 (95% CI 0.3–0.76)
[29]	Japan	2010	Case–control	Cases (n = 405) Controls (n = 2025) Age 18+	Vitamin D	Intakes ≥162 IU/day confer protection OR 0.64 (95% CI 0.43–0.94)
[21]	Korea	2010	Case–control	Cases (n = 144) Controls (n = 288) Age 18+	Vitamin A	Total intakes of vitamins A were strongly inversely associated with cervical cancer risk: OR = 0.35 (95% CI 0.19–0.65).
[24]	Brazil	2010	Case–control	Control n = 453. Cases: - CIN 1- 2 (n = 186) - CIN3 (n = 231) IC (n = 108) Age 21–65, HIV−	Alpha- Tocopherols Gamma- Tocopherols	Increasing levels of serum a–tocopherol: CIN2: OR 0.45 (%95 CI 0.25–0.81) CIN3: OR 0.26 (%95 CI 0.15–0.47) Increasing levels of serum g-tocopherol: CIN3: OR 0.46 (%95 CI 0.29–0.73)
[16]	USA	2007	Case–control	Controls (n = 979)Cases (n = 239) Age 21–59	Vitamin AVitamin E	Vit A intake of >12.7 IU/day–CC: OR = 0.47; (95% CI 0.3–0.73) Vit E intake of >8.9 mg/day–CC: OR = 0.44 (95% CI 0.27–0.72)
[18]	India	2002	Case–control	- Cases (n = 30) - Controls (n = 30) Age 35–55	Vitamin E	Vitamin E plasma levels are lower in cases vs. controls
[25]	USA	1997	Prospective cohort	n = 123 Ages 18+. Low-income	Alpha-tocopherols	Serum concentrations were lower among women two times HPV+. Independent of HPV status, lower serum levels correlated with higher grade of cervical dysplasia Normal 21.57 uM/CIN 1 21.18 uM/CIN 2 18.10 uM/CIN 3 17.27 u
[20]	USA	1992	Pilot case–control	Cases (n = 58) Controls (n = 42) Age 18+	Vitamin A (retinol)	Low retinol intake is associated with an increased risk of CIN 1 Q1 vs. Q4: OR = 2.3, (95% CI 1.3–4.1).

Table abbreviations: confidence interval (CI); carcinoma in situ (CIS); food frequency questionnaire (FFQ); hazard ratio (HR); invasive squamous cervical cancer (ISC); odds ratio (OR); quartile (Q); risk ratio (RR); correlation coefficients (r). Values reflect different study designs and are not directly comparable. * Europe refers to the countries that participated in the EPIC cohort: France, Germany, Greece, Italy, The Netherlands, Spain, and the United Kingdom.

#### 3.2.3. Minerals

Most minerals function as cofactors in cell differentiation and play essential roles in the immune system. A case–control study in Japan found that calcium intake above 502 mg/day may offer protection against CC [29] (Table 3). Additionally, a study in the United States reported that calcium intake was significantly associated with a 17% decrease in HPV infection risk [30]. Another study showed that calcium supplementation was significantly associated with a reduced risk of CIN2/3 progression [31].

Adequate iron levels also appear important for CC prevention [10]. A Brazilian study from the Ludwig–McHill cohort—one of the largest longitudinal studies of HPV and CC risk [32]—showed that HPV clearance was less likely in women with iron serum levels above the median (≥120 µg/L) (HR = 0.73, 95% CI 0.55–0.96) [33].

Zinc (Zn) has also been identified as relevant in cervical pathogenesis. A strong correlation was found between zinc intake and the prevention of CC or lesion progression [10]. A cross-sectional study in Italy observed an inverse relationship between Zn intake and hrHPV infection, suggesting immunomodulatory properties [34]. A clinical trial in Iran showed that Zn supplementation (zinc sulfate tablets twice daily for 3 months) in HPV-positive women led to higher rates of HPV clearance and cervical pathology regression [35].

**Table 3:** Studies analyzing the effect of minerals on HPV infection and CC risk and/or progression, by date.

Ref.	Country	Year of Publication	Study Design Data Collection	Sample Size Population Age (years) HIV/HPV+	Mineral	Results
[10]	Iran	2023	Population-based cross-sectional study. (MLM)	n = 2088 Mean age 34	Iron (Fe) Zinc (Zn) Potassium (K) Copper (Cu)	Strong correlation between mineral intake and a preventive effect regarding CC phase progression Fe: −0.671/−0.678 (r) Zn: −0.678/−0.731 (r) K: −0.574/−0.725 (r) Cu: −0.602/−0.731 (r)
[35]	Iran	2022	Randomized clinical trial	n = 80 (40 controls, 40 cases) Ages 21–55, HPV+	Zinc	Oral Zn sulfate associated with higher rates of HPV clearance and regression of cervical pathology. OR 0.13 (CI 95% 0.04–0.381)
[36]	USA	2021	Cross-sectional study	n = 4628 Age 16–59	Zinc Copper	Zn intake (Q4 vs. Q1)–hrHPV: OR 0.72 (95% CI 0.54–0.98) Cu intake (Q3 vs. Q1)–hrHPV: OR 0.67 (95% CI, 0.50–0.90) Zn intake (RDA established vs. below RDA)–hrHPV OR: 0.74; (95% CI 0.60–0.92)
[30]	USA	2020	Secondary analysis	n = 13,475 Age 18–59	Calcium (Ca)	Dietary Ca intake (log2) significantly associated with a 17% lower risk of HPV OR 0.83 (95% CI 0.70, 0.98)
[34]	Italy	2020	Cross-sectional study.	n = 251 Age 18+	Zinc	Zn negatively associated hrHPV risk (*p* < 0.001). Immunomodulatory properties.
[37]	Nigeria	2019	Case–control	- Controls (n = 45) - CIN cases (n = 45) Age ≤ 65	Selenium (Se)	Serum levels different between different CIN grades (*p* = 0.021). Linear trend (*p* = 0.025) Se can be used as cofactor to modulate HPV progression to cCC.
[33]	Brazil	2013	Cohort study (Ludwig–McHill)	n = 327 Age 18–60, HPV+	Iron	Clearance was less likely in women whose Fe serum levels were above the median. HR = 0.73 (95% CI 0.55–0.96) Rising Fe stores (≥120 ug/L) may decrease the probability of clearing a new HPV infection. HR = 0.34 (95%CI 0.15–0.81)
[29]	Japan	2010	Case–control	- Cases (n = 405) - Control (n = 2025) Age 18+	Calcium	≥502.6 mg/day confer protection. OR 0.5; 95% CI = 0.35–0.73
[31]	Japan	2010	Cross-sectional study	n = 1096 Age 18–65	Calcium	Ca supplements significantly associated with a lower risk of CIN 2/3. OR 0.21 (95% CI (0.08–0.50)

Table abbreviations: confidence interval (CI); carcinoma in situ (CIS); food frequency questionnaire (FFQ); hazard ratio (HR); invasive squamous cervical cancer (ISC); Recommended dietary allowance (RDA) odds ratio (OR); quartile (Q); risk ratio (RR); correlation coefficients (r). Values reflect different study designs and are not directly comparable.

#### 3.2.4. Other Nutrients

Carotenoids, which are pigments convertible into vitamin A in the human body [19], have demonstrated potential in CC prevention. A case–control study conducted in China found higher carotenoid concentrations in control subjects, suggesting an association with reduced CC risk [38] (Table 4). Another case–control study reported that increasing total serum carotenoid levels correlated with a lower risk of CIN3 (OR: 0.39) and invasive cancer (OR: 0.19) [24]. Effective daily doses linked to reduced CC risk were reported to be >1393 μg of α-carotene and >7512 μg of β-carotene [16].

Lycopene, a carotenoid without vitamin A activity that is found in tomatoes [39], was also shown to reduce CC risk. A Brazilian case–control study showed that higher serum lycopene levels were associated with decreased risk of CIN3 and invasive cancer [24], and daily intakes greater than 5837 μg were linked to reduced CC risk [16].

Polyphenols such as quercetin and genistein were found to have potentially beneficial effects. These compounds, often classified as antioxidants [40], may provide supportive benefits in cases of CC requiring chemotherapy. Preliminary evidence suggests that quercetin [41] and genistein [42] may have synergistic effects when used as complementary agents.

**Table 4:** Studies analyzing the effect of different nutrients on HPV infection and CC risk and/or progression, by date.

Ref.	Country	Year of Publication	Study Design Data Collection	Sample Size Population Age(years)HIV/HPV+	Nutrient	Results
[8]	USA	2021	Cohort (NHANES)	n = 11,070 Age 18–59	Albumin, Nutritional Antioxidant Score (NAS) (Include VitA, B2, E, B9)	Lowerserum albumin levels–increased risk of hrHPV <39 g/L OR 1.4 (95%CI 1.1–1.) Higher NAS associated with lower odds of hrHPV infection. Low vs. high NAS OR 1.3 (1–1.7); lrHPV: Low vs. high NAS OR 1.4 (1.1–1.7)
[38]	China	2015	Hospital-based case–control	Cases (n = 200) Controls (n = 158) Age 18+	α-carotene β-carotene Lutein Tocopherols	High concentrations of carotenoids and tocopherols associated with low CC risk α-carotene OR 0.42 (0.26, 0.66) β-carotene OR 0.31 (0.20, 0.47)Lutein OR 0.53 (0.35, 0.79) (*p* 0.003) - ocopherols OR 0.39 (0.26, 0.58)
[24]	Brazil	2010	Case–control	Control n = 453 - CIN 1- 2 (n = 286) - CIN3 (n = 231) - Invasive cancer (n = 108) Age 21–65, HIV−	Lycopene Carotenoids	Increasing levels serum lycopene decrease CIN 3 [OR 0.43 (%95 CI 0.27–0.68)], invasive cancer [OR 0.17 (%95 CI 0.08–0.35)]. Increasing total levels of serum carotenoids decrease the risk of CIN 3 [OR 0.39 (%95 CI 0.25–0.62)], invasive cancer [OR 0.19 (%95 CI 0.09–0.38)]
[16]	USA	2007	Case–control	Controls (n = 979) - Cases (n = 239) Age 21–59	Fiber α-carotene β-carotene Lutein Lycopene	>29 g fiber/day—OR = 0.59; (95% CI 0.37–0.94) ↓ risk of CC >1.393 μg α-carotene/day—OR = 0.41 (95% CI 0.27–0.63) ↓ risk of CC >7.512 μg β-carotene/day—OR = 0.44; (95%CI 0.29–0.68) ↓ risk of CC >6558 μg lLtein/day—OR = 0.51 (95% CI 0.33–0.79) ↓ risk of CC >5.837 μg lycopene/day—OR = 0.65 (95% CI 0.44–0.98) ↓ risk of CC

Table abbreviations: low (↓), odds ratio (OR); quartile (Q); confidence interval (CI); NutrAntioxidant Score (NAS). Values reflect different study designs and are not directly comparable.

#### 3.2.5. Foods

Aside from specific nutrients, the intake of fruits and vegetables has been shown to play an important role in preventing CC. A lower intake is associated with a higher risk of hrHPV infection [43] (Table 5). Specifically, a daily intake of less than 109 g/day has been shown to increase the risk of developing CIN2 and CIN3 in hrHPV-positive women with a high viral load [43] and consuming less than 319 g/day is associated with progression to CIN3 stages [44]. Ultimately, a daily intake of 100 g/day was inversely associated with invasive squamous cell cervical carcinoma [17].

A lower intake of vegetables is associated with an increased risk of CC [45]. An intake below 302 g/day is linked to progression to CIN2 and CIN3 stages in women with a high viral load, and a higher risk of progression to CIN3+ stages in women with cervical lesions [43]. Consuming 100 g/day of vegetables is associated with decreased risk of invasive squamous cell cervical carcinoma [17], and intake over 207 g/day reduces the risk of HPV persistence [46]. Diets richer in fruits and vegetables are also linked to a lower risk of hrHPV infection and cervical lesion progression [24,43].

**Table 5:** Studies analyzing the effect of different foods on HPV infection and CC risk and/or progression, by date.

Ref.	Country	Year of Publication	Study Design Data Collection	Sample Size Population Age HIV/HPV+/−	Food	Results
[10]	Iran	2023	Population-based cross-sectional study. (MLM)	n = 2088 Mean age 34	Dairy products	Strong positive correlation with CC. Yogurt (r = 0.778), Milk (r = 0.775)
[23]	USA	2023	Cohort (NHANES)	n = 11,070 Age 18–59	Fruits Whole fruits Greens and beans	Lower intake of these is associated with hrHPV infection. Fruit intake for women with hrHPV infection vs. no hrHPV: 2.5 to 5 pieces a day: (Fruits/whole fruits/greens and beans) 95% CI OR 0.61 (0.45–0.85)/OR 0.57 (0.42–0.78)/OR 0.61 (9.47–0.80) More than 5 pieces a day: OR 0.57 (0.42–0.78)/OR 0.62 (0.47–0.81)/OR 0.68 (0.55–0.83)
[45]	China	2012	Matched case–control (ratio 1:9)	- Cases (n = 102) - Controls (n = 963) Diagnosis of CIN 2, 3 or CC. Age 28–61	Vegetables	A higher intake of fresh vegetables could decrease the risk of CC OR 0.89 (95% CI 0.81–0.99)
[17]	Europe *	2010	Prospective cohort study	n = 299,651 ISC (n = 253). CIS (n = 817) Age 35–70	Fruits Vegetables Leafy vegetables	Consumption of 100 g is inversely associated with ISC Fruits: HR 0.83; 95% CI 0.72–0.98 Vegetables: HR 0.85: 95% CI 0.65–1.10 Higher consumption of leafy vegetables is associated with a lower risk of developing invasive squamous cell CC. HR = 0.52 (95% CI 0.29–0.95)
[47]	Brazil	2010	Cohort study (Ludwig–McHill)	n = 327 Age 18–60, HPV+	Fruit	Orange consumption ≥1 time/week decreases the risk of squamous intracellular lesions for HPV+ women. OR 0.32 (95% CI 0.12–0.87)
[48]	China	2001	Cross-sectional population-based study	n = 2338 - Normal cervix (n = 2143) - CIN2 + (n = 195) Age 35–50	Onion Legumes Nuts Meat	Intake of >15.95 servings per week is associated with a lower risk of the development of CIN+. OR = 0.65 (95% CI 0.44–0.988) Intake of >2.69 servings per week is associated with a lower risk of the development of CIN+. OR = 0.65; (95% CI 0.44–0.98) Intake of >0.61 servings per week is associated with a lower risk of the development of CIN+. OR = 0.59; (95% CI 0.39–0.88) Intake of >0.94 servings per week is associated with a lower risk of the development of CIN+. OR = 0.65; (95% CI 0.43–0.99)
[24]	Brazil	2009	Case–control	Control n = 453 - CIN 1- 2 (n = 286) - CIN3 (n = 231) - Invasive cancer (n = 108) Age 21–65, HIV−	Carrots	203–1321 g carrots/day protective CIN3: OR 0.46 (%95 CI 0.31–0.70).
[43]	Korea	2010	Cohort study	n = 1096 Age 18–65 y	Fruits Vegetables	Low fruit intake (<109 g/d) in women with high viral load for hrHPV infection is associated with a higher risk of developing CIN 2/3 compared to women with a decreased viral load. OR = 2.93 (95% CI 1.25–6.87) Low vegetable intake (<302 g/d) in women with a high viral load for hrHPV infection: increase in risk of CIN 2/3 compared to those with a lower viral load. OR 2.84 (1.26–6.42)
[44]	Brazil	2009	Hospital-based case–control	- Control n = 453 - CIN1 (n = 140) - CIN2 (n = 126) - CIN3 (n = 231) - Invasive cancer (n = 108) Ages 21–65, HIV−	Green and yellow produce Fruit and juice Fruits and vegetables	Consumption of ≤39 g/d green and yellow produce is associated with CIN 3. OR = 1.71 (95% CI = 1.15–2.52) Consumption of ≤79 g/d of fruit and juice is associated with CIN 3. OR = 1.44 (95% CI = 1.02–2.03) Consumption of ≤319 g/d of fruits and vegetables is associated to CIN 3. OR = 1.52 (95% CI 1.06–2.17)
[46]	USA	2002	Prospective cohort study	n = 1042 Age 18–35	Vegetables	Higher consumption of vegetables (>207 g/day) is associated with decreased risk of HPV persistence. OR 0.46 (0.21–0.97)

Table abbreviations: confidence interval (CI); carcinoma in situ (CIS); food frequency questionnaire (FFQ); hazard ratio (HR); invasive squamous cervical cancer (ISC); odds ratio (OR); quartile (Q); risk ratio (RR); correlation coefficients (r). Values reflect different study designs and are not directly comparable. * Europe refers to the countries that participated in the EPIC cohort: France, Germany, Greece, Italy, The Netherlands, Spain, and the United Kingdom.

#### 3.2.6. Functional Foods

Probiotics have recently been assessed for their efficacy in treating CC [49] (Table 6). *Lactobacillus crispatus* has shown effectiveness in promoting HPV clearance by modulating the intravaginal microbiota, reducing populations of *Gardnerella* and *Prevotella* [50]. Other *Lactobacillus* species and some *Bifidobacterium* have demonstrated anticancer activity against CC and have been beneficial in mitigating the side effects of chemotherapy [51]. Additionally, an in vivo study with mice in Iran found that the intravenous or oral administration of *Bifidobacterium bifidum* modulated the immune response through IL-12 and IFN-gamma, preventing the growth of HPV-induced tumors [52].

**Table 6:** Studies analyzing the effect of functional foods on HPV infection and CC risk and/or progression, by date.

Ref.	Country	Year of Publication	Study Design Data Collection	Sample Size Population Age HIV/HPV+	Functional Food	Results
[50]	China	2024	Controlled pilot study	n = 100 - Lactobacillus (n = 50) - Placebo (n = 50) Age 18–65, R- hrHPV+	Probiotics *Lactobacillus crispatus* (*L. crispatus*)	Intravaginal probiotics (*L. crispatus*) were successful for the following: - Effective HPV clearance: Probiotics vs. placebo 12.13% higher (*p* > 0.05). - Cytological improvement rate: 82.14% vs. 77.78%, both *p* < 0.05. - Significantly improved vaginal microbiota, with a downward trend in *Gardnerella* and *Prevotella* (*p* < 0.01).
[51]	India	2024	Review	Age 30–69, CC diagnosis	Probiotics *Lactobacillus* *Bifidobacterium*	Some *Lactobacillus* and *Bifidobacterium* strains have shown anticancer activities against CC and were found to be helpful in combating side effects

#### 3.2.7. Specific Diets and Dietary Patterns

Dietary patterns such as the Mediterranean Diet (MD), which emphasizes antioxidant-rich foods like fresh fruits and vegetables, have been associated with a lower risk of CC (Table 7). A study in Italy found that adherence to the MD was linked to a reduced risk of hrHPV infection, and adherence to the Prudent diet was associated with lower risk of CIN2+ in HPV-positive women [53]. In Colombia, a conservative dietary pattern—including vegetables, fruits, whole foods, and supplements—was linked to a lower incidence of CC in women aged 35–64 years [54]. Conversely, diets with high glycemic load (GL) were significantly associated with increased risk of CIN1 [55], and a pro-inflammatory dietary pattern was linked to increased risk of CIN2 or more severe cervical lesions [7].

**Table 7:** Studies analyzing the effect of different dietary patterns on HPV infection and CC risk and/or progression, by date.

Ref.	Country	Year of Publication	Study Design Data Collection	Sample Size Population Age HIV/HPV+	Dietary Pattern	Results
[54]	Colombia	2023	Multi-group ecological study	n = 3472 Age 35–64	Conservative pattern	This is related to a lower incidence of CC.
[7]	Italy	2022	Cross-sectional study	n = 539 Age 18+	Pro-inflammatory diet	High adherence to this diet-increased risk of CIN2 or severe lesions. OR 3.14 (95% CI 1.50–6.56).
[55]	Korea	2020	Case–control	n = 1340 Age 18–65	High glycemic load	Diets with high glycemic load significantly associated with CIN1 risk: OR 2.8 (95% CI 1.33–5.88).
[53]	Italy	2018	Cross-sectional study	Age 18+. n = 539 - Normal cervical epithelium (n = 252) - CIN1–2 (n = 217) - CIN3+ (n = 70)	Mediterranean diet (MD) Prudent diet	Medium adherence to MD linked to a lower risk of hrHPV infection. OR = 0.43 (95% CI 0.22–0.73). Adherence to Prudent diet is protective against development of CIN 2+. OR = 0.50 (95% CI 0.26–0.98).

Table abbreviations: confidence interval (CI); Mediterranean Diet (MD); odds ratio (OR); quartile (Q). Values reflect different study designs and are not directly comparable.

## 4. Discussion

### 4.1. Main Findings

A broad spectrum of vitamins, minerals, food groups, dietary patterns, and bioactive non-nutrient compounds has demonstrated potential in the prevention and management of cervical carcinogenesis. As previously noted, persistent infection with HPV remains a principal risk factor for the development of CC [8]. Certain nutrients have been shown to facilitate HPV clearance, thereby reducing the likelihood of progression to precancerous or cancerous lesions. Others may contribute to the regression of CIN at various stages. Furthermore, specific dietary components and functional foods may offer supportive benefits during CC treatment, including the mitigation of therapy-related adverse effects. However, some inconsistencies across studies remain, which may be explained by differences in intake levels, population characteristics, or study design.

Although these findings are encouraging, additional research is warranted to elucidate underlying mechanisms and to refine their integration into evidence-based prevention strategies and therapeutic approaches.

#### 4.1.1. Water-Soluble Vitamins

The relationship between water-soluble vitamin intake and the development and progression of CC and its precursors was analyzed. The findings were consistent with the previous literature indicating that micronutrients such as B-complex vitamins and vitamin C play crucial roles in immune function, DNA synthesis, and oxidative stress regulation—factors that influence HPV persistence and cervical carcinogenesis.

The observed inverse associations between vitamin B1 supplementation and HPV infection, and between folate levels and CIN progression, align with previous studies conducted in both high- and low-resource settings. For example, earlier research has shown that folate deficiency may enhance the carcinogenic potential of HPV through impaired DNA repair mechanisms. Similarly, studies have consistently reported that antioxidant vitamins such as vitamin C contribute to epithelial cell protection and immune surveillance, supporting our findings. It is important to consider that a significant proportion of the studies included are observational in nature, which limits the ability to infer causality between specific nutrients and CC prevention. While antioxidants, folate, and carotenoids have been frequently associated with a lower risk of CC, the limited number of randomized clinical trials reduces the strength of the evidence and prevents definitive conclusions regarding their protective effects.

#### 4.1.2. Liposoluble Vitamins

Vitamins A, D, and E may play a protective role in the development and progression of CC. Their inverse associations with CIN and HPV infection suggest they could contribute to lowering disease risk. These vitamins are thought to have antioxidant properties, although the extent and mechanisms of their antioxidant effects remain unclear [12,56].

This antioxidant potential might underlie their influence on immune response and cellular health, which could explain their role in reducing CIN persistence and progression. Vitamin D, in particular, can modulate immune responses by regulating the activity of immune cells, such as T lymphocytes. However, further studies are needed to confirm these mechanisms and their implications in CC prevention.

#### 4.1.3. Minerals

Minerals such as calcium, iron, and zinc appear to influence cervical carcinogenesis through immune modulation and cellular homeostasis. While some studies suggest a protective effect of calcium [16,31], others report an increased risk of HPV infection with higher intake [10], highlighting the complexity of its role and the possibility of dose-dependent or population-specific effects. Iron levels must be carefully regulated; although essential [10], excess iron may hinder HPV clearance, as shown in large-scale cohort data [33]. Zinc stands out for its consistent association with HPV clearance and lesion regression [10,17,34,35], likely due to its immunomodulatory and antiviral properties. These findings emphasize the importance of mineral balance in CC prevention, though further studies are needed to clarify optimal intake levels and mechanisms of action.

#### 4.1.4. Other Nutrients

The findings suggest that non-vitamin nutrients, such as carotenoids, lycopene, and polyphenols, may contribute to the prevention or management of CC. Carotenoids appear to play a protective role, likely due to their antioxidant capacity and role in modulating cellular oxidative stress [16,24,38]. Lycopene, although not contributing to vitamin A synthesis, shows a similar protective trend, particularly in later stages of lesion development [16,24]. Polyphenols, especially quercetin and genistein, offer potential as adjunctive agents during cancer treatment due to their antioxidant and possibly cytotoxic effects [40-42]. While the preliminary evidence is promising, further studies are needed to clarify mechanisms, optimal dosing, and potential integration into therapeutic protocols.

#### 4.1.5. Foods

Overall, a higher intake of fruits and vegetables has a protective effect against the development of cervical lesions and CC. This protective role is likely related to their content of vitamins, antioxidants, and other bioactive compounds that support immune function and inhibit disease progression.

#### 4.1.6. Functional Foods

These findings suggest that probiotics may serve as a promising tool for CC prevention and adjunctive therapy. Their potential lies in immune modulation and microbiota regulation. However, further research—particularly in high-prevalence populations—is needed to fully validate these effects and guide clinical application.

#### 4.1.7. Specific Diets and Dietary Patterns

These findings suggest that healthy dietary patterns rich in fruits, vegetables, and antioxidants may help reduce the risk of cervical lesions and cancer [51,52]. In contrast, diets with a high glycemic load or pro-inflammatory properties may contribute to disease progression [53,54]. Promoting balanced, anti-inflammatory dietary habits could therefore be an effective strategy for CC prevention.

### 4.2. Limitations

Nutritional strategies for CC prevention have demonstrated promising associations between dietary patterns and a reduced risk of disease; however, several important limitations remain. This review was limited to studies indexed in PubMed, meaning relevant studies available in other databases may have been missed. A significant proportion of the included studies are observational in nature, which limits the ability to infer causality between specific nutrients and CC prevention. While antioxidants, folate, and carotenoids have been frequently associated with a lower risk of CC, the limited number of randomized clinical trials reduces the strength of the evidence and prevents definitive conclusions regarding their protective effects.

Moreover, the reliance on self-reported dietary intake in many of these studies introduces the possibility of recall bias and reporting inaccuracies, which complicates the interpretation of results. Dietary intake is also inherently influenced by various confounding factors, including physical activity levels, socioeconomic status, and cultural practices, all of which vary significantly across populations. These differences limit the generalizability of findings and hinder the isolation of effects attributable to individual nutrients. In addition, genetic and environmental variability—as well as inconsistencies in global dietary recommendations—can affect nutrient metabolism, bioavailability, and their overall impact on cancer risk.

Several studies also face methodological constraints such as small sample sizes and short follow-up durations, limiting their capacity to assess long-term outcomes of dietary interventions. Importantly, while nutrition may contribute to enhanced immune function and improved HPV clearance, persistent infection with hrHPV types remains the primary etiological factor in CC. As such, dietary strategies cannot substitute for established preventive interventions, including HPV vaccination and regular CC screening.

Future research should prioritize large-scale, randomized controlled trials to establish evidence-based dietary recommendations. These studies should also incorporate assessments of nutrient ADME (absorption, distribution, metabolism, and excretion), bioavailability, and interactions between dietary components and other preventive measures, including vaccination and screening programs. 

## 5. Conclusions

Overall, maintaining a varied diet rich in fresh fruits and vegetables appears to contribute to CC prevention at multiple stages. This protective effect is likely due to the high content of vitamins, minerals, antioxidants, and other bioactive compounds present in these foods. The findings presented in this review support the notion that nutrition is a modifiable risk factor for CC. Specifically, dietary habits may help prevent persistent HPV infections and facilitate viral clearance.

In terms of cervical intraepithelial neoplasia (CIN), nutritional interventions have been associated with a potential to halt lesion progression. Certain vitamins—most notably folate and vitamin C—play critical roles in preventing the advancement of CIN, with vitamin C showing a dose-dependent protective effect. Practical implementation should consider achievable dietary sources, recommended intake limits, and integration into routine clinical care to ensure safety and effectiveness. Adequate mineral intake is also essential, given its contribution to reducing the risk of HPV infection, lesion progression, and CC development.

Functional foods, particularly probiotics, have demonstrated promise in enhancing HPV clearance and supporting a balanced cervical microbiome. Antioxidants are especially important due to the broad spectrum of compounds within this group. Among them, polyphenols have shown potential in CC treatment by enhancing the efficacy of chemotherapy through synergistic mechanisms.

Nonetheless, further research is required to reinforce these findings and establish more definitive conclusions. Strengthening the evidence base will support the formal integration of nutritional strategies into future prevention and treatment guidelines. A comprehensive approach that combines dietary interventions with established preventive measures—such as HPV vaccination and regular screening—may improve CC outcomes and reduce the global burden of this disease, particularly in low- and middle-income countries.

## Data Availability

No new data were created or analyzed in this study. Data sharing is not applicable to this article.

## Abbreviations

The following abbreviations are used in this manuscript:

CC	Cervical cancer
CIN	Cervical intraepithelial neoplasia
CIS	Carcinoma in situ
GL	Glycemic load
HPV	Human Papillomavirus
hrHPV	High-risk Human Papillomavirus
IGF	Insulin-like growth factor
ISC	Invasive squamous cervical cancer
lrHPV	Low-risk Human Papillomavirus
MD	Mediterranean diet
NAS	Nutritional Antioxidant Score
SSA	sub-Saharan Africa
STI	Sexually transmitted infection
